# *In Vitro* and *In Vivo* Attenuation of Vesicular Stomatitis Virus (VSV) by Phosphoprotein Deletion

**DOI:** 10.1371/journal.pone.0157287

**Published:** 2016-06-17

**Authors:** Phonphimon Wongthida, Juggragarn Jengarn, Jaraspim Narkpuk, Pongpisid Koonyosying, Kanjana Srisutthisamphan, Asawin Wanitchang, Pornsawan Leaungwutiwong, Samaporn Teeravechyan, Anan Jongkaewwattana

**Affiliations:** 1 Virology and Cell Technology Laboratory, National Center for Genetic Engineering and Biotechnology (BIOTEC), 113 Thailand Science Park, Phahonyothin Rd., Klong Nueng, Klong Luang, Pathum Thani, 12120, Thailand; 2 Protein-Ligand Engineering and Molecular Biology Laboratory, National Center for Genetic Engineering and Biotechnology (BIOTEC), 113 Thailand Science Park, Phahonyothin Rd., Klong Nueng, Klong Luang, Pathum Thani, 12120, Thailand; 3 Department of Biochemistry, Faculty of Science, Kasetsart University, Ngam Wong Wan Rd., Ladyaow, Chatuchak, Bangkok, 10900, Thailand; 4 Department of Microbiology and Immunology, Faculty of Tropical Medicine, Mahidol University, Ratchawithi Rd., Ratchadewee, Bangkok, 10400, Thailand; Chang Gung Memorial Hospital, TAIWAN

## Abstract

Vesicular stomatitis virus (VSV) is highly immunogenic and able to stimulate both innate and adaptive immune responses. However, its ability to induce adverse effects has held back the use of VSV as a potential vaccine vector. In this study we developed VSV-ΔP, a safe yet potent replication-defective recombinant VSV in which the phosphoprotein (P) gene was deleted. VSV-ΔP replicated only in supporting cells expressing P (BHK-P cells) and at levels more than 2 logs lower than VSV. *In vivo* studies indicated that the moderate replication of VSV-ΔP *in vitro* was associated with the attenuation of this virus in the mouse model, whereas mice intracranially injected with VSV succumbed to neurotoxicity. Furthermore, we constructed VSV and VSV-ΔP expressing a variety of antigens including hemagglutinin-neuraminidase (HN) from Newcastle disease virus (NDV), hemagglutinin (HA) from either a 2009 H1N1 pandemic influenza virus (pdm/09) or the avian H7N9. VSV and VSV-ΔP incorporated the foreign antigens on their surface resulting in induction of robust neutralizing antibody, serum IgG, and hemagglutination inhibition (HAI) titers against their corresponding viruses. These results indicated that VSV with P gene deletion was attenuated *in vitro* and *in vivo*, and possibly expressed the foreign antigen on its surface. Therefore, the P gene-deletion strategy may offer a potentially useful and safer approach for attenuating negative-sense RNA viruses which use phosphoprotein as a cofactor for viral replication.

## Introduction

Vesicular stomatitis virus (VSV) belongs to the family *Rhabdoviridae* in the genus *Vesiculovirus*. VSV is an enveloped, bullet-shaped virus with a negative, single-stranded, 11,161-bp RNA genome encoding 5 proteins: nucleocapsid (N), phosphoprotein (P), matrix protein (M), glycoprotein (G) and large RNA-dependent RNA polymerase (L) [1,2]. Infectious VSV can be efficiently recovered by a reverse genetics approach that utilizes multiple plasmids expressing VSV genes [3,4]. Since the development of the VSV infectious clone, VSV has been considered a vaccine vector candidate due to its inability to undergo genetic recombination and genetic reassortment, and the absence of transforming properties. Furthermore, recombinant VSV can accommodate large or multiple foreign gene inserts in its genome, and has been used to express a variety of viral antigens [5–10]. These recombinants elicit strong humoral and cell-mediated immune responses upon use as vaccines and also mediate protection against their own virus challenge in mice and non-human primates. Along with the ability to generate robust immune responses, VSV naturally infects at mucosal surfaces, enabling the use of intranasal immunization to induce both mucosal and systemic immunity [11].

Natural VSV infection can cause disease in cattle, horses and swine, resulting in symptoms such as vesicular lesions around the mouth, hoofs, and teats [2,12]. Natural infection of humans is rare and has only been reported in agricultural or veterinary contexts [13,14]. There has also been a report of a laboratory worker infected after direct exposure to VSV [15]. Although human infection is typically subclinical or only results in mild flu-like symptoms [14,16], a single case of encephalitis in a Panamanian boy associated with VSV Indiana infection has been documented [17]. Neurotropism and neurovirulence have also been demonstrated in experimental VSV infection of rodents [18–20].

Based on the neurological effects, research objectives have shifted towards developing VSV with decreased virulence for use as a human vaccine vector [7,8,21–24]. These attenuated VSV can undergo a single or multiple rounds of infection and viral transcription, however, possibly inducing neurotoxicity. We have therefore developed a novel strategy to eliminate viral transcription and strongly reduce the risk of VSV neurotoxicity by deleting the P gene in the VSV genome (VSV-ΔP). This P gene is known to encode an essential cofactor responsible for an active polymerase complex. Deletion of the P gene should completely abrogate transcription of VSV in infected normal cells while allowing for host immune response activation, leading to a safer yet equally effective vaccine compared to wild-type VSV. We have been able to show that VSV-ΔP replicated strictly in supporting cells and caused no neurovirulence in mice. More importantly, we demonstrate that VSV-ΔP expressing hemagglutinin-neuraminidase (HN) from Newcastle disease virus (NDV) or hemagglutinin (HA) from a 2009 H1N1 pandemic influenza virus (pdm/09) or the avian H7N9 were able to incorporate HN or HA on their surface. Moreover, mice immunized with these recombinant viruses generated robust immune responses similar to their replication-competent counterparts.

## Materials and Methods

### Cell lines and plasmids

Baby hamster kidney (BHK)-21 and BHK-21 cells expressing phosphoprotein (P) of VSV (BHK-P) were grown in DMEM (Pan Biotech, Germany) supplemented with 10% fetal bovine serum (FBS). Madin-Darby canine kidney (MDCK) and human embryonic kidney (HEK)-293T cells were grown in Opti-MEM (Invitrogen, USA) supplemented with 10% FBS.

pUbEm, pCMV-ΔR8.91 and pMD.G were kindly provided by Dr. Yasuhiro Ikeda (Mayo Clinic, USA) [25] and pVSV-XN2 by Dr. John Rose (Yale University, USA).

### Influenza viruses and reverse genetics

H1N1 influenza viruses A/Puerto Rico/8/34 (PR8) and A/Nonthaburi/102/2009 (pdm/09) were prepared from embryonated chicken eggs. Recombinant H7N9 was constructed as previously described [26] using the HA and NA genes from A/Shanghai/02/2013 (accession number KF9188659.1) in the background of PR8. The recombinant viruses were rescued and propagated as previously described [27].

### Generation of the BHK-P cell line

To construct a lentiviral vector containing the P gene of VSV, the P gene was amplified from pVSV-XN2 using P gene-specific primers. The PCR product was inserted into pUbEm to generate pUbEm-P. Lentivirus harboring the P gene was generated based on the method described previously [25] and used to transduce BHK-21 cells. Single clones of BHK-P cells were obtained by limiting dilution.

### Generation of recombinant VSV-based constructs

**Fig 1:**
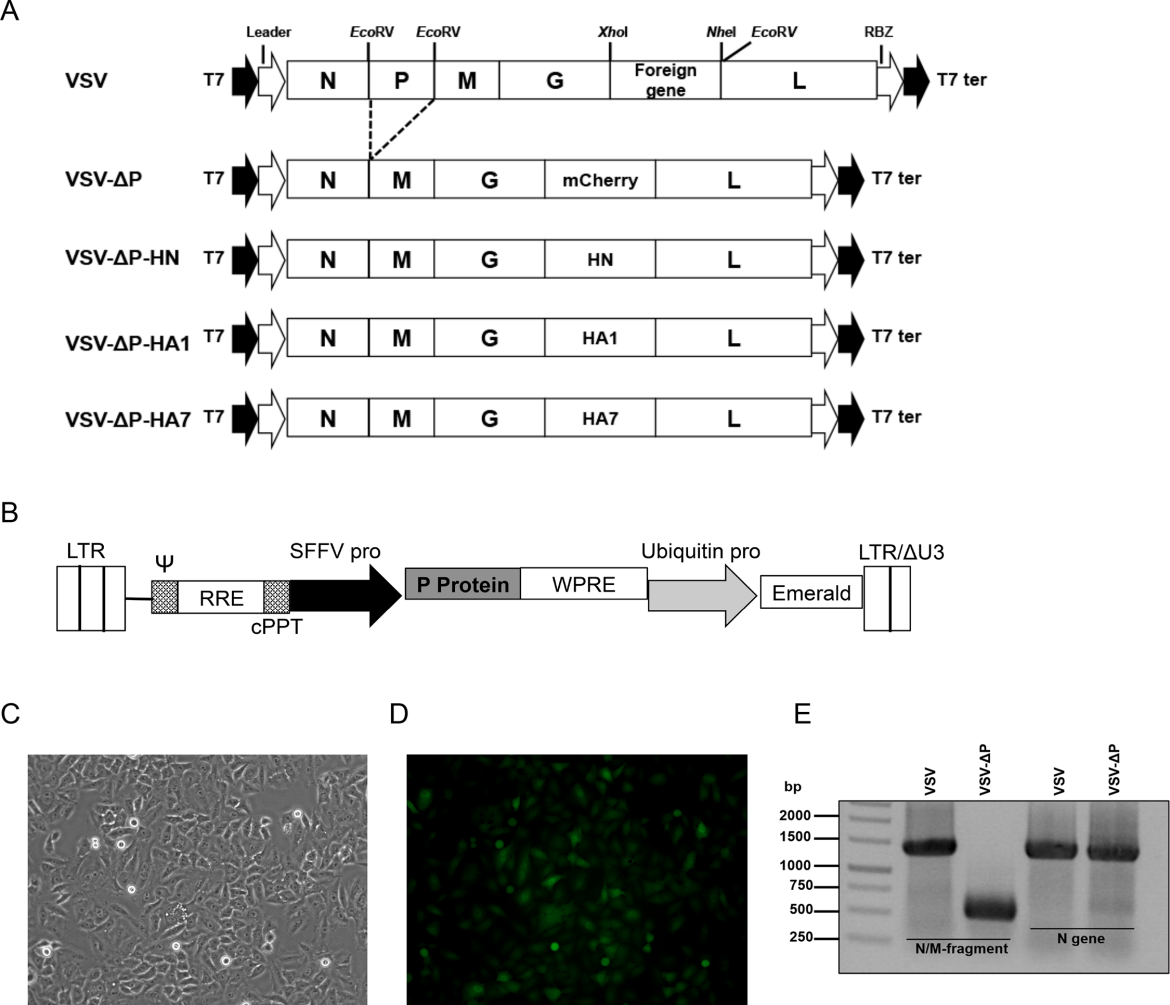
Construction of recombinant VSVs with P gene deletion. (A) The top schematic shows the VSV genome layout and the naturally occurring restriction sites used for cloning. To construct VSV-ΔP, the VSV genome was digested with *Eco*RV, religated in the absence of the P gene fragment and the mCherry gene was then inserted between the G and L genes. The mCherry gene was replaced by HN, H1 or H7 HA to generate VSV-ΔP-HN, VSV-ΔP-HA1 and VSV-ΔP-HA7, respectively. (B) Supporting cells (BHK-P) were constructed by transducing BHK-21 cells with lentivirus bearing the P gene, and the selected clone expressing the emerald fluorescent protein was examined by (C) bright field and (D) fluorescence imaging. (E) BHK-P cells were infected with VSV or VSV-ΔP, and supernatants were harvested for viral genome extraction and RT-PCR. RBZ, hepatitis virus delta ribozyme; T7, T7 RNA polymerase leader; T7 ter, T7 terminator; LTR, long terminal repeat; ψ, packaging signal; RRE, rev responsive element; cPPT, central polypurine tract; SFFV, spleen focus-forming virus (promoter); WPRE, woodchuck hepatitis virus post-transcription regulatory element; ΔU3, U3 deletion.

pVSV-XN2, which encodes the genome of VSV Indiana serotype, was digested with *Eco*RV (Fig 1A). The P gene fragment was removed and the rest of the VSV genome was self-ligated. pVSV-ΔP was constructed by inserting the mCherry gene from pmCherry-C1 (Clontech, USA) into *Xho*I and *Nhe*I sites. Similarly, pVSV-ΔP-HN, pVSV-ΔP-HA1 and pVSV-ΔP-HA7 were generated by insertion of HN (amplified from NDV strain LaSota), pdm/09 H1 HA (amplified from A/Nonthaburi/102/2009 RNA) and H7 HA (synthesized by Bio Basic Inc, Canada, based on A/Shanghai/02/2013 (H7N9) HA), respectively. For construction of pVSV-HN, pVSV-HA1 and pVSV-HA7, HN, H1 or H7 HA genes were inserted into the original pVSV-XN2 at the same position. All VSVs were then recovered based on the method described previously [3,28]. Bulk amplification of plaque-purified VSVs were subsequently performed by infecting either BHK-21 cells with VSVs or BHK-P cells with VSV-ΔPs at an MOI of 0.01 for 24 h. Filtered supernatants were centrifuged twice in a 10% sucrose cushion at 27,000 rpm (Sorvall T-880 fixed-angle rotor) for 1 h at 4°C. Pelleted viruses were re-suspended in PBS, aliquoted and stored at -80°C. VSV stocks were titrated on BHK-21 or BHK-P cells using the standard plaque assay [29].

### Flow cytometry

BHK-21 and BHK-P cells (5×10^5^ cells/well) were seeded overnight before inoculation with VSV-ΔP at an MOI of 1 for 1 h at 37°C, and then washed once with PBS. Expression of mCherry and emerald proteins in infected cells was photographed at 48 h post-infection using a fluorescence microscope. Subsequently, cells were harvested, fixed in 4% formaldehyde and subjected to flow cytometry analysis using the Cytomics FC 500 MPL (Beckman Coulter, USA). Percentages of infected cells were calculated from the ratios of mCherry-expressing cells to total cell number.

### Growth curves

BHK-P cells were infected with either VSVs or VSV-ΔPs at an MOI of 0.01 for 1 h at 37°C, prior to washing with PBS. Supernatants were collected at 6, 12, 24, 48 and 72 h post-infection. Virus titers were determined by the standard plaque assay.

### SDS-PAGE and Western blot analysis

To detect expression of HA on the viral surface, 1×10^7^ purified VSV-HA1 and VSV-ΔP-HA1 were lysed in radio-immunoprecipitation assay buffer supplemented with 1% ProteoBlock Protease Inhibitor Cocktail (Thermo Scientific, USA), and the proteins were electrophoretically separated in a 10% sodium dodecyl sulfate–polyacrylamide gel. HA and VSV-G proteins were detected by Western blotting using a polyclonal antibody against pdm/09 HA (Sino Biological, China) and serum from VSV-immunized mice, respectively.

### Reverse transcription polymerase chain reaction (RT-PCR)

vRNA was extracted from the supernatant of cells infected with VSVs or VSV-ΔPs using the Viral Nucleic Acid Extraction Kit II (Geneaid, Taiwan). Viral cDNA synthesis was performed with the RevertAid Premium first-strand cDNA synthesis kit (Thermo Scientific). To distinguish between viral RNA obtained from VSVs and VSV-ΔPs, primers for cDNA synthesis were designed to bind to the end of N gene (forward primer 5’ CAGCTCTTCTGCTCAGATCCACCAG 3’) and beginning of the M gene (reverse primer 5’ TTCAGACCGAGAATCTTCTTTAAGGAACTC 3’). H7 HA (5’ATCTCGAGATGAACACTCAAATCCTG 3’ as the forward primer and 5’ ATGCTAGCTTATATACAAATAGTGCACC 3’ as the reverse primer) and VSV-N (5’ GCTGATCAATGTCTGTTACAGTCAAGAG 3’ as the forward primer and 5’ ATGCGGCCGCTCATTTGTCAAATTC 3’ as the reverse primer) were detected using specific primers.

### *In vivo* studies

All procedures were approved by the Animal Care and Use Committee, Faculty of Tropical Medicine, Mahidol University, Thailand. BALB/c and ICR mice were purchased from the National Laboratory Animal Center, Mahidol University. For vaccine safety, five 3–4 week-old ICR mice per group were lightly anesthetized with ether and then intracranially injected with 1×10^4^ pfu of virus. Mouse health and body weight were monitored daily for 14 days. Animals exhibiting neurological symptoms (limping, paralysis, etc.) were euthanized according to institutional guidelines and were recorded as showing a lethal response. To study immune induction, five 6–8 week-old BALB/c mice per group were intravenously injected with 1×10^7^ pfu of virus at days 0 and 21, and were monitored daily after vaccination. At day 28, sera were harvested and tested for immune response induction.

### Hemagglutination inhibition (HAI) assay

Sera were 2-fold serially diluted with PBS in U-bottom, 96-well microtiter plates (Thermo Scientific). Four HA units of influenza virus were added into each well and incubated at room temperature for 1 h followed by addition of 0.75% human type-O red blood cells. HAI titer was calculated as the reciprocal of the highest dilution of serum which completely inhibited the agglutination of red blood cells.

### Microneutralization assay

Sera from immunized mice were 2-fold serially diluted in 96-well plates before being mixed with 100 TCID_50_ of each influenza virus. The virus–antibody mixtures were incubated at 37°C for 1 h and added to monolayers of MDCK cells seeded in 96-well plates. Plates were incubated at 37°C for 1 h then washed, and media with 2 μg of TPCK-trypsin were added to each well. Plates were incubated at 37°C for another 72 h and supernatants were analyzed by the HA assay. Virus neutralization titer was defined as the reciprocal of the highest dilution of serum which showed completely no HA titer [30].

### Indirect ELISA for HA-specific IgG in serum

Ninety-six-well microtiter plates were coated with two HA units of influenza virus diluted in bicarbonate coating buffer (pH 9.6) and blocked with 10% FBS. Serially diluted sera were added to the wells, and HA-specific IgG detected with HRP-conjugated goat anti-mouse IgG (KPL, USA). Tetramethyl benzidine substrate was added for readout at OD_450_ with a Multiskan FC ELISA plate reader (Thermo Scientific).

### Statistical analysis

Survival data from the animal studies were analyzed with the log-rank test using GraphPad Prism 4 (GraphPad Software, USA). Statistical significance of the data was determined by Student’s t-test.

## Results

### 1. Engineering the VSV genome by P gene deletion

We hypothesized that manipulating genes in the VSV genome to prevent virus replication would impair the virus’ ability to induce neurotoxicity. To first engineer the attenuated VSV, we excised the P gene from the VSV genome with *Eco*RV, which recognizes sequences before the P gene start codon, after the M gene intergenic region and before the L gene (Fig 1A). Three of the fragments, minus the P gene fragment, were self-ligated and the mCherry gene was inserted between the G and L genes, generating VSV-ΔP (Fig 1A). To rescue and propagate VSV-ΔP, we constructed BHK-21 cells stably expressing P (BHK-P) by lentiviral transduction (Fig 1B–1D). Rescued viruses were confirmed for P gene deletion by RT-PCR of viral RNA (Fig 1E), thereby demonstrating successful VSV-ΔP construction and replication in the supporting BHK-P cells.

### 2. P gene deletion disabled replication of recombinant viruses in normal cells

**Fig 2:**
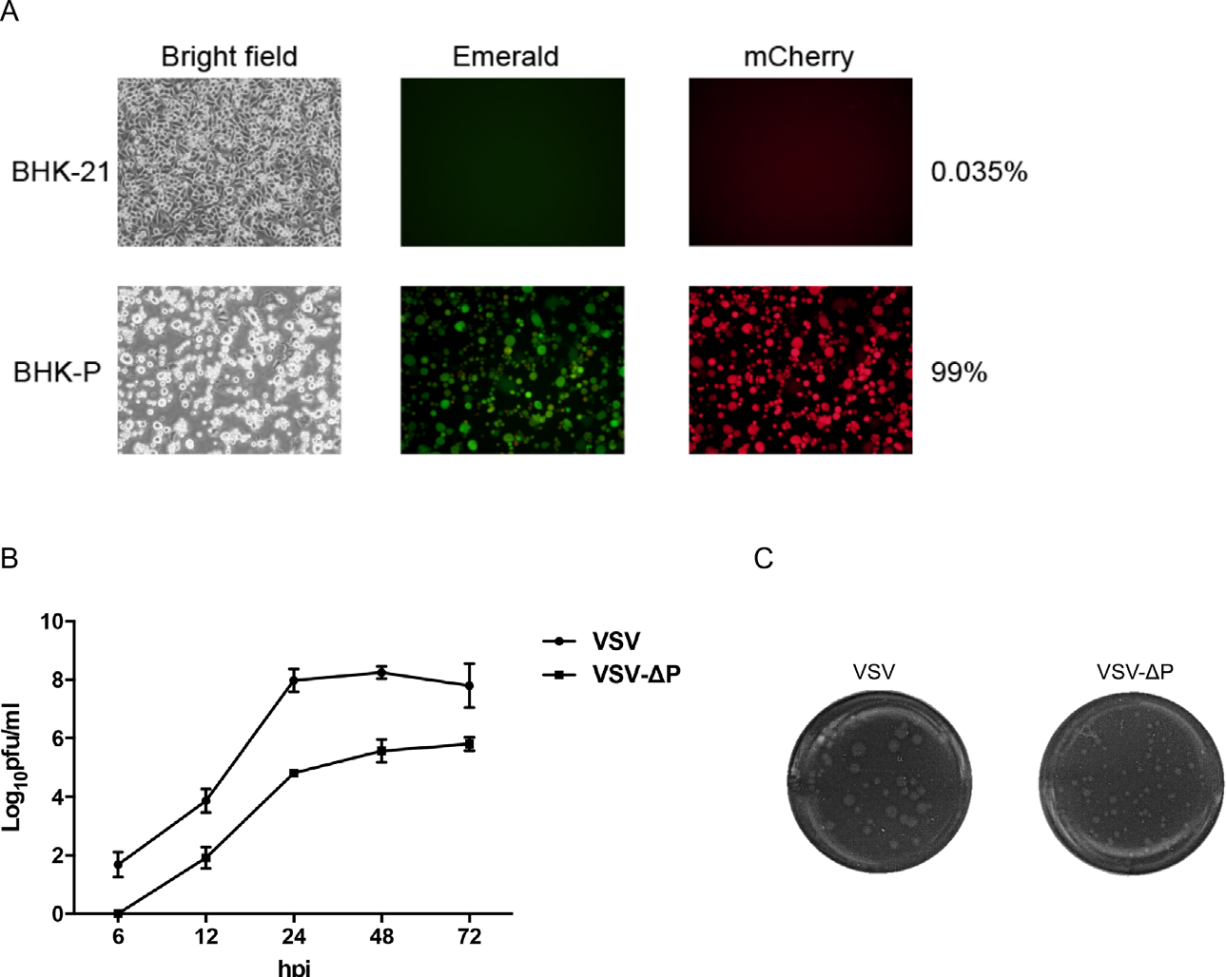
P gene deletion attenuated replication of recombinant virus. (A) BHK-21 and BHK-P cells were infected with VSV-ΔP at an MOI of 1 and observed for cytopathic effects (CPE). Infected cells were then subjected to flow cytometry to quantify the percentage of mCherry-expressing cells. The pictures are representative of triplicate samples. (B) BHK-P cells were infected with VSV or VSV-ΔP at an MOI of 0.01. Supernatants were harvested at the indicated time points for plaque assays. Values are averages of two independent experiments with error bars showing standard deviation (SD). (C) Viruses were serially diluted for plaque titration, and plaques were stained with neutral red for visualization. Representative images of VSV and VSV-ΔP were selected for plaque size comparison.

To demonstrate that P gene deletion abrogates the ability of VSV to replicate in normal cells, we examined expression of emerald (BHK-P cells) and mCherry (VSV-ΔP-infected cells) proteins by flow cytometry. As expected, VSV-ΔP exhibited impaired replication in normal BHK-21 cells with less than 0.05% fluorescent cells detected among infected cells (Fig 2A). In contrast, almost 100% of BHK-P cells were infected. To investigate the effect of P gene deletion on viral replication, VSV-ΔP and VSV were used to infect BHK-P cells at an MOI of 0.01 and supernatants were harvested at various time points. In contrast to VSV, VSV-ΔP was first observed 12 h post-infection, and remained at least 2 logs lower than VSV at all time points (Fig 2B). Consistent with viral replication, VSV-ΔP produced smaller plaques than VSV (Fig 2C), and the plaques were detected 48 h post-infection compared to 24 h post-infection for VSV. These data indicated that the P-gene deletion substantially impaired virus growth *in vitro*.

### 3. Intracranial injection demonstrated decreased neurovirulence of VSV-ΔP

**Fig 3:**
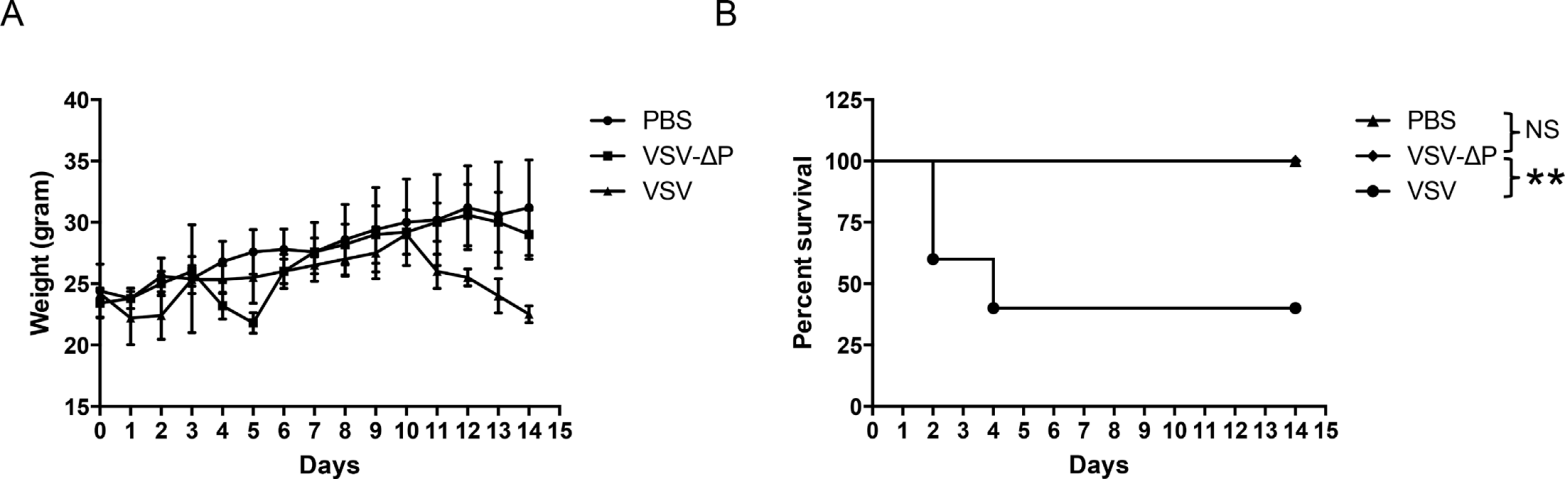
Decreased lethality in mice after intracranial injection with VSV-ΔP. ICR mice (5 mice/group) were lightly anesthetized with ether and then intracranially injected with either PBS or 1×10^4^ pfu of VSVs. (A) Body weight was measured daily and (B) survival was plotted using the Kaplan-Meier survival curve. Values are averages of five mice with error bars showing SD and are representative of two independent experiments. NS, not significant; *, p<0.5; **, p<0.05.

Thus far, we have shown VSV-ΔP attenuation (Fig 2B and 2C) and restriction *in vitro* (Fig 2A). To investigate replication and attenuation *in vivo*, mice were intracranially injected with 1×10^4^ pfu of the VSVs, and observed for neurological symptoms daily. Mice injected with VSV began limping on day 3 post-injection and started losing weight at day 2, with more pronounced weight loss at day 14 (p = 0.03). In contrast, mice injected with VSV-ΔP had no significant weight loss, similar to mice injected with PBS (Fig 3A). Although the body weight of mice injected with VSV-ΔP was reduced on day 5, possibly from mild neuropathogenic effects of virus injection, no neurological symptoms were otherwise observed. Moreover, the survival rate of mice injected with VSV-ΔP was significantly greater than those injected with VSV (p = 0.0486) (Fig 3B), where >50% either died or were euthanized following neurological symptoms within 3 days. In contrast, all mice injected with VSV-ΔP survived and showed no outward signs of neurovirulence.

### 4. VSVs expressed foreign antigens on the viral surface

**Fig 4:**
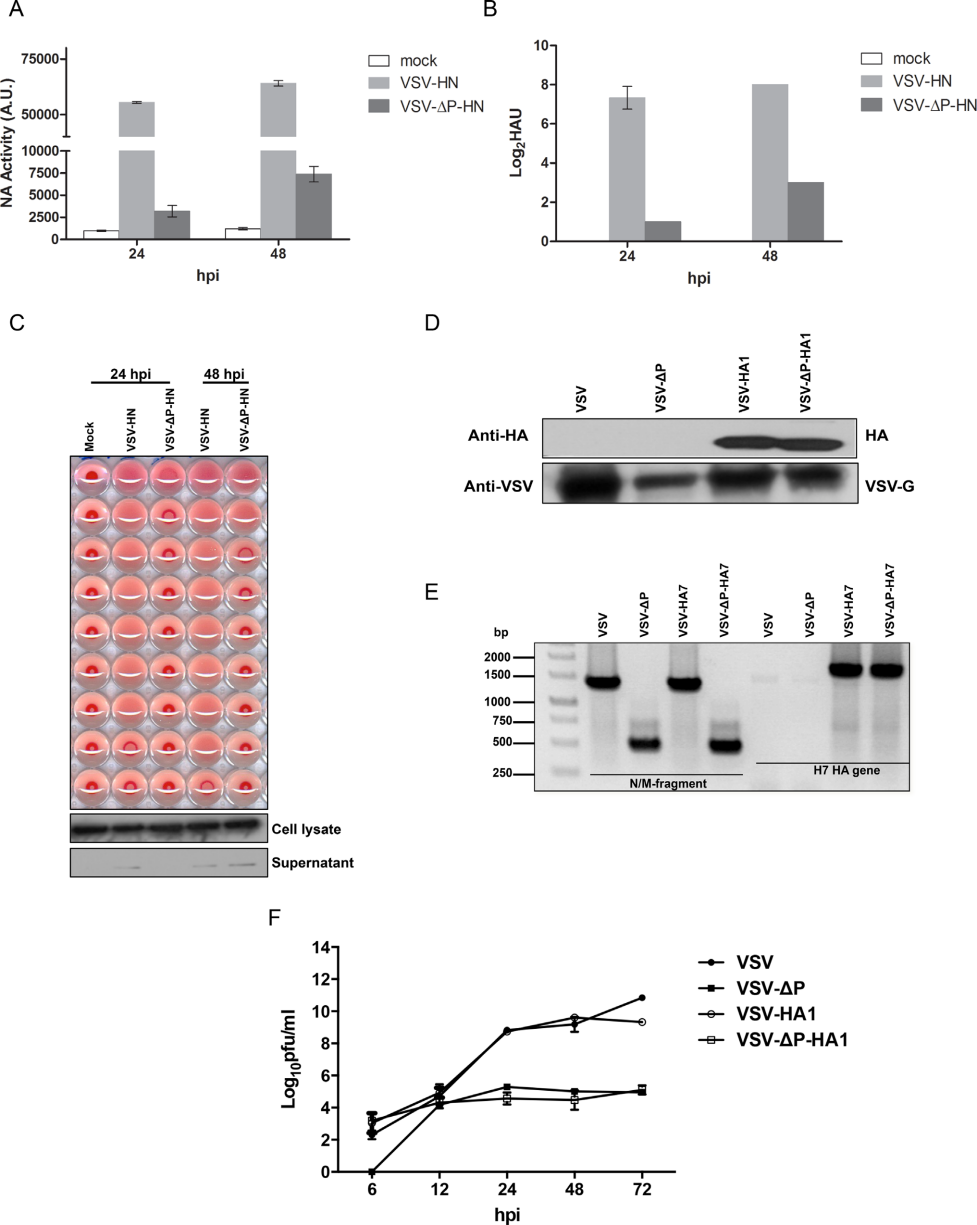
Expression of foreign antigens on the viral surface. (A) BHK-P cells were infected with VSVs at an MOI of 0.1. Supernatants were harvested at the indicated time points and were assessed using a MUNANA-based assay, (B) HA assay and (C) HAI assay. Supernatant and cell lysates were subjected to Western blot analysis using a β-actin monoclonal antibody as the primary antibody. Values are averages of triplicate wells with error bars showing SD. (D) 1×10^7^ purified VSVs were lysed and subjected to Western blot analysis using an HA (H1N1) polyclonal antibody and serum from VSV-immunized mice as the primary antibody. (E) To study the incorporation of the H7 HA gene in VSVs, RNA were extracted from purified VSVs and subjected to RT-PCR using primers specific for the N/M fragment or H7 HA genes. (F) BHK-P cells were infected with VSVs at an MOI of 0.01. Supernatants were harvested at the indicated time points for plaque assays. Values are averages of two independent experiments with error bars showing SD. A.U., arbitrary units.

To test the attenuated VSV-ΔP as a vaccine vector candidate, we constructed VSV and VSV-ΔP expressing HN from NDV (LaSota strain) or HA from a 2009 H1N1 pandemic influenza virus (pdm/09) and the avian A/Shanghai/02/2013 (H7N9). VSV and VSV-ΔP expressing HN (VSV-HN and VSV-ΔP-HN, respectively; Fig 1A) incorporated HN on their viral surface as demonstrated by NA activity (Fig 4A) and the ability to inhibit red blood cell agglutination by HA assay (Fig 4B and 4C). NA activity was detected as early as 24 h post-infection and reached the highest point at 48 h (Fig 4A).

To demonstrate the expression of HA on VSV and VSV-ΔP expressing H1N1 HA (VSV-HA1 and VSV-ΔP-HA1, respectively; Fig 1A), purified viruses were analyzed for HA expression by Western blot analysis. While HA was not detectable on VSV or VSV-ΔP, both VSV-HA1 and VSV-ΔP-HA1 expressed HA, most likely on the viral surface (Fig 4D) [7]. Due to the lack of a commercial anti-H7 HA antibody, we checked for the incorporation of HA-encoding RNA in VSV and VSV-ΔP expressing H7N9 HA (VSV-HA7 and VSV-ΔP-HA7, respectively; Fig 1A) by RT-PCR. We found that VSV-HA7 and VSV-ΔP-HA7 contained the HA gene in their viral genomes and had the P gene deletion (Fig 4E).

Incorporation of foreign glycoproteins onto the viral surface suggested that the presence of HN or HA did not block viral assembly. To further examine whether insertion of a foreign gene interfered with virus replication, virus growth of VSV-HA1 and VSV-ΔP-HA1 was measured over time. All VSVs could be detected at 6 h except VSV-ΔP (Fig 4F), similar to Fig 2B. Replication of VSV-ΔP and VSV-ΔP-HA1 was 2–4 logs lower than the P-containing VSVs, with the difference becoming noticeable after 12 h. Taken together, incorporation of foreign glycoproteins did not disrupt viral replication compared to the respective parental strain, and these viruses expressed foreign glycoproteins on the viral surface, which is relevant for eliciting immune responses against surface antigens.

### 5. Immunization with VSV-ΔP expressing HA induced immune responses

**Fig 5:**
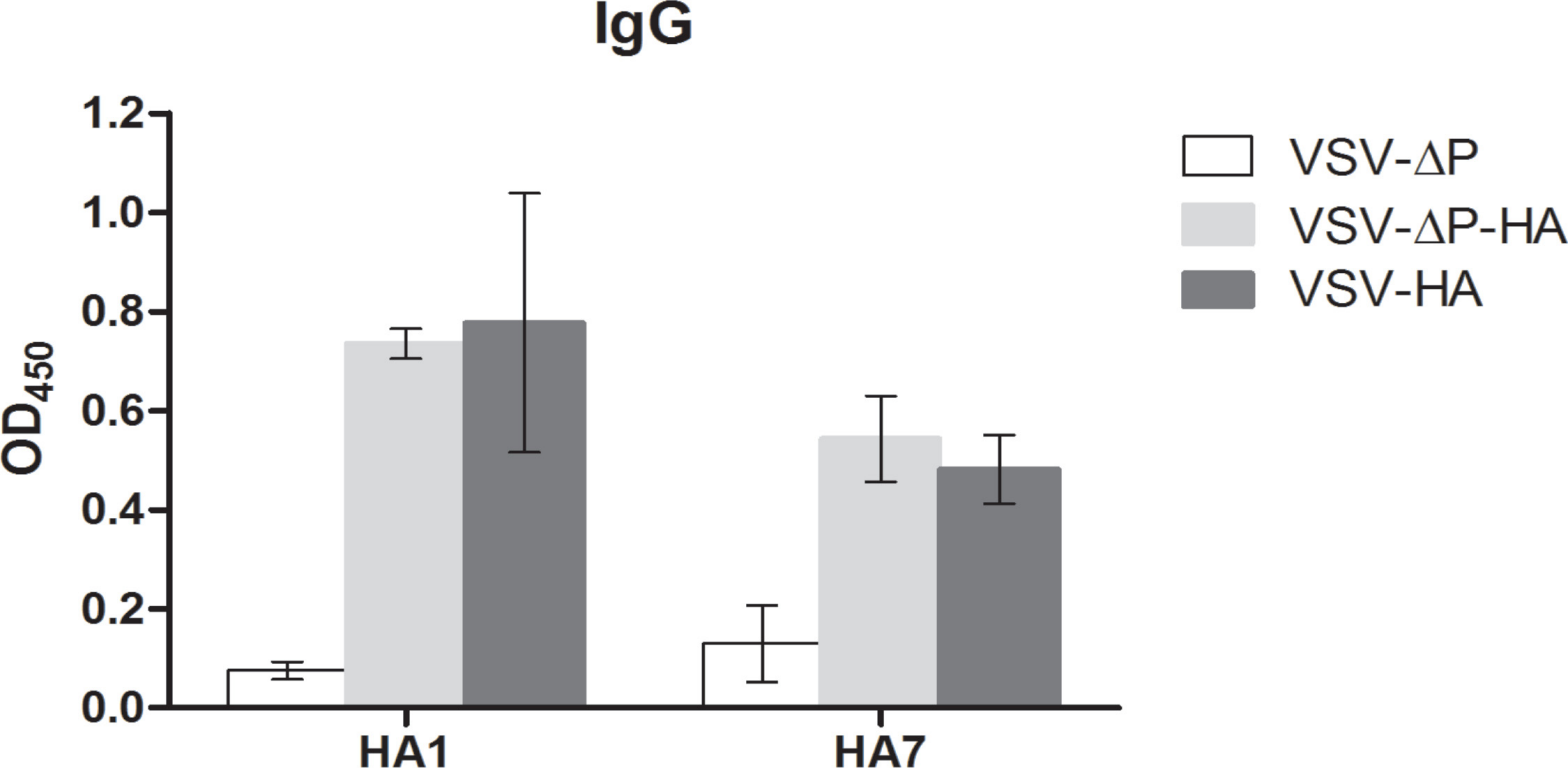
Induction of immune responses following immunization with VSV-ΔP expressing H1 and H7 HA. BALB/c mice (5 mice/group) were intravenously injected with 1×10^7^ pfu of VSVs in 100 μl at days 0 and 21. At day 28, sera were harvested to determine for H1N1-specific and H7N9-specific IgG levels at a titer of 5,120. Values are averages of two independent experiments with error bars showing SD.

Finally, we tested whether non-replicating VSV-ΔP could induce immune responses to the foreign antigen HA. Mice immunized with VSV-ΔP-HA1 or VSV-HA1 induced anti-HA IgG production in serum at titers of 5,120, which were significantly greater than those of mice immunized with VSV-ΔP (Fig 5). Moreover, these VSVs also induced neutralizing antibodies and notable HAI titers against HA from pdm/09. As shown in Table 1, mice immunized with VSV-ΔP-HA1 or VSV-HA1 generated virus neutralizing antibody titers at 160–640 and HAI titers at 1,280–2,560 and 640–2,560, respectively, against the pdm/09 strain A/Nonthaburi/102/2009 (H1N1) as well as other pdm/09 strains (data not shown). However, cross-reactivity was not observed against heterologous influenza viruses such as PR8 and H7N9.

**Table 1:** Virus neutralizing (VN) assay and hemagglutination inhibition (HAI) assay.

Vaccine group	pdm/09		H7N9		PR8	
	VN	HAI	VN	HAI	VN	HAI
**VSV-ΔP**	0	0	0	0	0	0
**VSV-ΔP-HA1**	160–640	1280–2560	0	0	0	0
**VSV-HA1**	160–640	640–2560	0	0	0	0
**VSV-ΔP-HA7**	0	0	160–320	320–640	0	0
**VSV-HA7**	0	0	320–640	640	0	0

Values are representative of two independent experiments.

Similarly, sera from mice immunized with VSV-ΔP-HA7 or VSV-HA7 developed significant anti-HA IgG levels compared to those from control mice (Fig 5), as well as strong neutralizing antibody and HAI titers against H7 HA (Table 1). For mice immunized with VSV-ΔP-HA7, sera neutralizing antibody and HAI titers were at 160–320 and 320–640, respectively, whereas those from mice immunized with VSV-HA7 were at 320–640 and 640, respectively. No cross-reactivity was found to either pdm/09 or PR8 (Table 1). These results demonstrate that immunization with VSV-ΔP expressing HA induced anti-HA IgG, neutralizing antibodies and HAI titers to the corresponding HA (Fig 5 and Table 1). The absence of P and the presence of G which might interfere with the immune induction, did not affect HA immune induction efficacy.

## Discussion

In this study, we successfully showed that manipulation of the VSV genome by deleting the phosphoprotein (P) gene (VSV-ΔP) attenuated viral replication *in vitro* and led to decreased pathogenicity in mice as monitored by weight loss and paralysis. The P gene itself encodes a multifunctional protein that plays a major role in polymerase activity by protecting L from proteolytic degradation [31], supporting efficient nascent RNA encapsidation by binding to newly synthesized N [32], and promoting viral RNA synthesis by interacting with the terminal sequence of the viral genome [33]. P gene disruption would therefore abrogate VSV replication and transcription, preventing viral protein expression and viral progeny production.

The generation of VSV-ΔP has been previously reported by Muik et al. (2012), where it was used as an oncolytic virus. They showed that VSV with the P gene deletion could not be propagated in normal BHK-21 cells, except by co-propagation of VSV-ΔP with VSV-ΔG or VSV-ΔL. The VSV-ΔP work was only briefly explored, and the characteristics of the virus, such as growth kinetics and titers or immune induction *in vivo*, were not described despite the very promising nature of its attenuation.

The most important aspect of attenuation by P gene deletion is the impact on viral transcription. While P is a structural protein, our data revealed no mCherry expression by VSV-ΔP-mCherry in BHK-21 cells even 48 hours after infection, suggesting that the P molecule packaged in each viral particle is insufficient for driving any appreciable level of protein expression. No sign of neuropathogenesis in mice intracranially injected with VSV-ΔP was observed either, due to restriction of VSV-ΔP replication to P-expressing cells. While we presume that no viral replication occurred in mice brain tissue, virus replication in such brain tissue needs to be further examined. Overall, VSV-ΔP appears to be replication-deficient both *in vitro* and *in vivo*.

On the other hand, other VSV attenuation strategies have not eliminated *in vitro* viral replication [22,23,34]. Schnell et al. (1998) reported VSV attenuation by a G cytoplasmic tail (CT) truncation, but this virus was still capable of replicating to high titers in cell culture. Similarly, the combination of methionine 51 deletion in the M gene and G CT-truncation yielded a titer of 5×10^3^ pfu/ml in a type I IFN-signaling-competent cell line [23]. Although no neurotoxicity was found in VSV-treated immune-competent mice, these viruses still possess an intrinsic ability to replicate. The possibility of *in vivo* replication cannot be entirely dismissed, especially in immune-compromised animals, and such replication may in turn present a risk of neuropathology.

P gene deletion is also practical for other reasons. First, with its relatively small size, the P gene can be deleted easily by common restriction enzymes without compromising the VSV genome. Furthermore, unlike other VSV proteins, cell lines stably expressing P is easy to generate, as stable expression of P has little effect on cell viability. In contrast, cells permanently expressing M have not been reported, presumably due to the association of M with some of the cytopathic effects of VSV infection, such as microtubule disassembly leading to cell rounding [35]. Similarly, as G is capable of mediating cell-cell fusion [36], cell lines stably and constitutively expressing G are simply not viable. An inducible promoter system has been reported [37], but the technique is complicated and technically challenging.

While VSV-ΔP replicated efficiently in BHK-P cells, its yield was still lower compared to its VSV counterpart. This difference was expected, as levels of P expression in the cell line were likely lower compared to the amount of P generated during wild-type virus infection. In addition, to function as a polymerase cofactor for viral RNA replication and transcription, P needs to be in phosphorylated form [38]. However, P in BHK-P cells might not be properly phosphorylated, and therefore may be less efficient in supporting VSV-ΔP replication. It may be possible to improve VSV-ΔP yields with strategies such as using stronger promoters, codon optimization or expressing phosphomimetic P. Nevertheless, VSV-ΔP yielded from BHK-P cells was comparable to that from the inducible G stable cell line, where VSV-ΔG expressing CD4 or CC4 was generated at titers in the range of 0.5×10^6^ to 1.0×10^6^ pfu/ml [37].

VSV-ΔP was shown to express H1 HA on the viral surface by Western blot analysis. However, we could not observe the hemagglutination activity of VSV-ΔP-HA (data not shown). We speculate that red blood cell binding may require the presence of both HA and NA, as NA has been shown to remove oligosaccharides adjacent to the HA receptor-binding pocket, thereby facilitating the binding between HA and sialic acid [39]. This was also observed in a previous study using VSV expressing HA of WSN influenza virus, where the virus was not able to bind red blood cells unless the cells used to generate the virus were treated with NA [40]. The observation that our VSV-HN and VSV-ΔP-HN viruses, which possess both hemagglutinin and neuraminidase function, exhibited hemagglutination activity strongly supports this theory.

VSV-ΔP-HA generated robust immune responses similar to its counterpart virus. The efficacy was presumably due to the capacity for single-round entry and by viral surface presentation of HA. Given that VSV-ΔP-HA expresses HA on its surface but is unable to replicate in normal cells, it likely behaves similar to a virus-like particle except for the presence of an RNA genome. Consequently, activation of Toll-like receptors, particularly TLR3 and/or TLR7, could have enhanced maturation of dendritic cells and cytotoxicity of natural killer cells [41], heightening immune responses against the foreign glycoproteins.

In summary, we demonstrate here that VSV-ΔP technology is a viable platform for virus attenuation. We believe that the P gene deletion strategy may be further applied to the attenuation of other non-segmented, negative-sense RNA viruses that use a phosphoprotein to replicate, such as rhabdovirus, paramyxovirus, and bornavirus.

## Abbreviations

VSV: vesicular stomatitis virus
P: phosphoprotein
HN: hemagglutinin-neuraminidase
HA: hemagglutinin
HAI: hemagglutination inhibition
